# Supplementary figures and images for: Altering lipid droplet homeostasis affects Coxiella burnetii intracellular growth
Source: PLoS One. 2018 Feb 1;13(2):e0192215. doi: 10.1371/journal.pone.0192215 (PMC5794150; doi:10.1371/journal.pone.0192215)

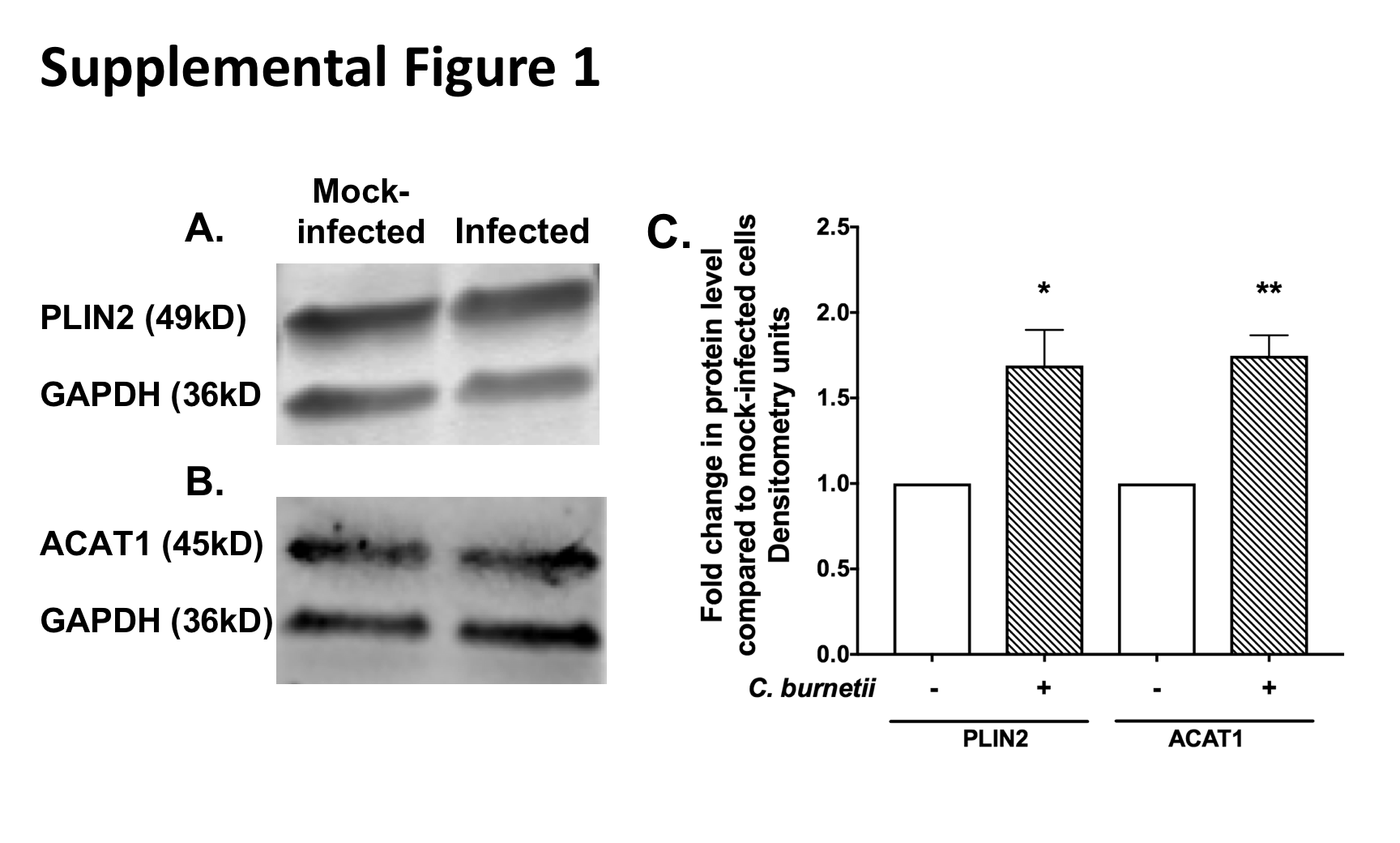

Supplement: S1 Fig — MH-S macrophages were infected with wild-type C. burnetii and cell lysates collected at day 2 post-infection were immunoblotted for (A) PLIN2 and (B) ACAT1. Shown is a representative blot from three separate experiments. (C) Protein levels were quantitated, normalized to GAPDH, and the fold change over mock-infected cells plotted. Error bars show the mean of 3 independent experiments +/- SEM * = p < .05, ** = p <0.01 compared to respective mock-infected as determined by unpaired t-test. (TIFF) [file pone.0192215.s001.tiff]

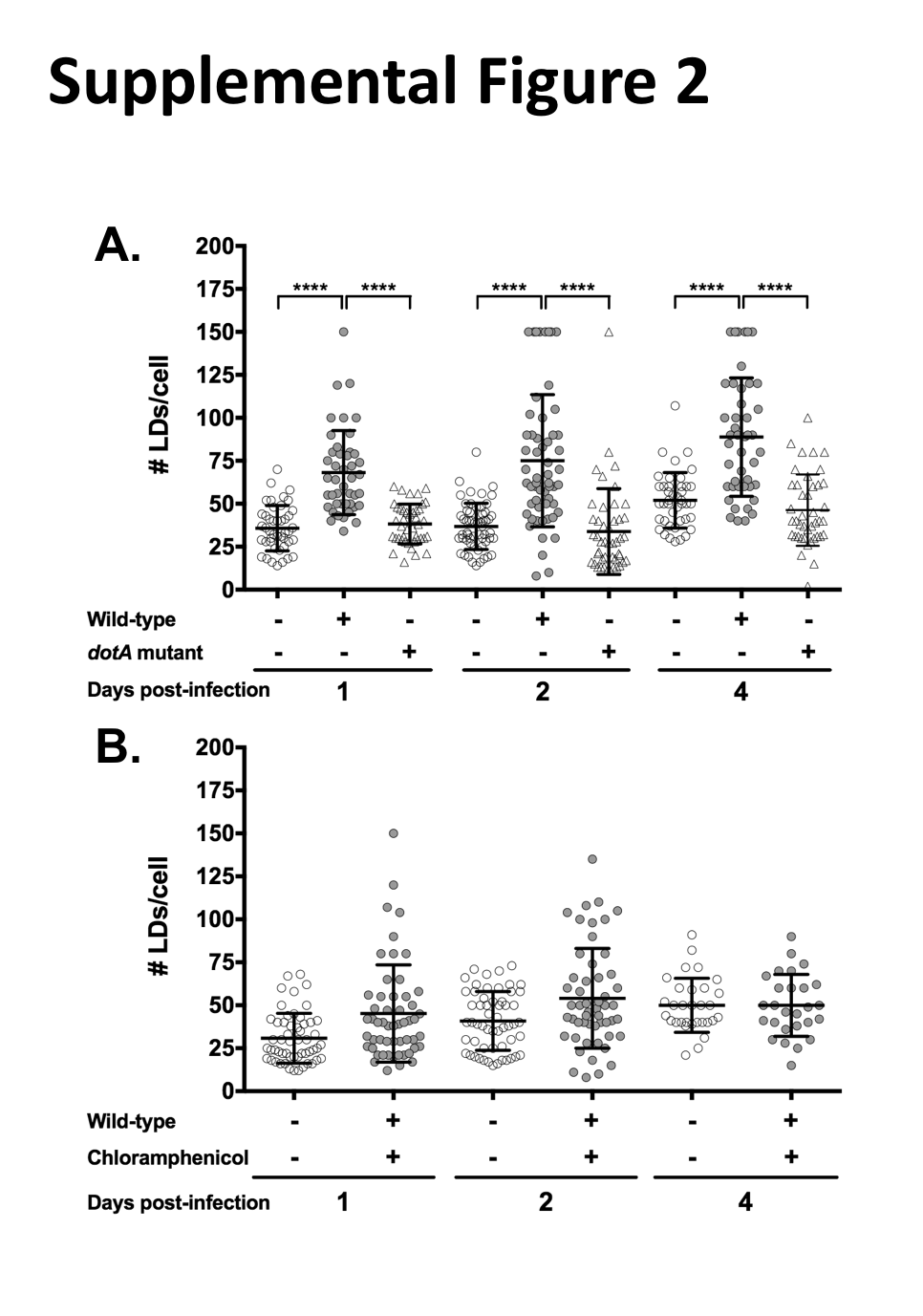

Supplement: S2 Fig — acat-1-/- macrophages were infected with C. burnetii and at different times post-infection, cells were stained for PLIN2, C. burnetii and nucleus. LD number per cell were quantitated by fluorescence microscopy. A) LD numbers in wild-type C. burnetii and dotA mutant- infected acat-1-/- macrophages. B) LD numbers in acat-1-/- macrophages infected with wild-type C. burnetii and treated with chloramphenicol (3ug/ml). Error bars show the mean of 3 independent experiments +/- SEM **** = p <0.0001 as determined by ordinary one-way ANOVA with Tukey post-hoc test. (TIFF) [file pone.0192215.s002.tiff]

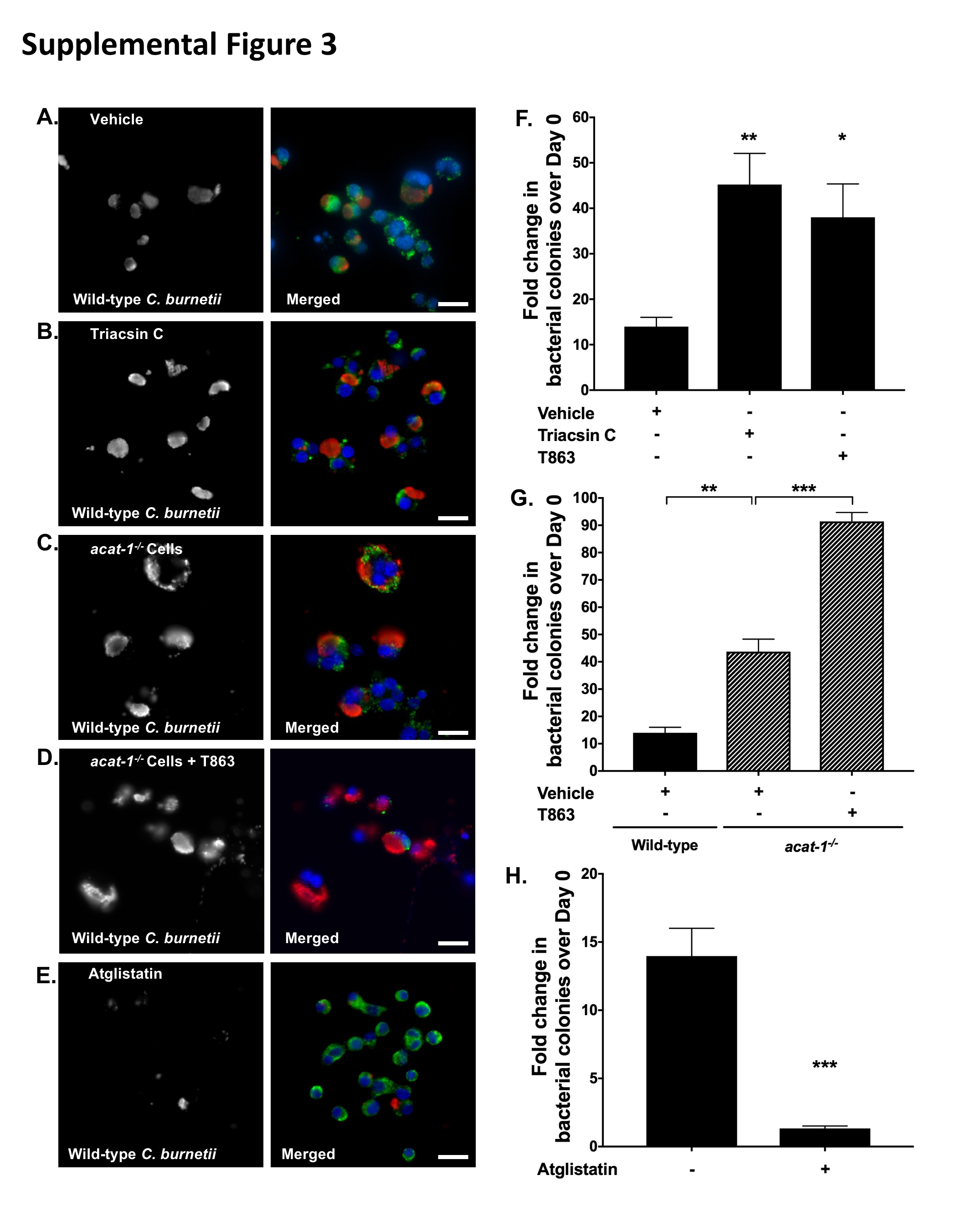

Supplement: S3 Fig — Wild-type C. burnetii growth in infected MH-S cells treated with different inhibitors was measured at day 4 post-infection by CFU assay. A-E) Representative images for wild-type MH-S macrophages treated with inhibitors, fixed, stained for PLIN2 (LDs; green) and C. burnetii (red) and imaged day 4 post-treatment at 20X oil. Scale bar = 20 µm. F) Growth while inhibiting LD formation with triacsin C (10 µM) and T863 (10 µM) in wild-type MH-S macrophages. Error bars represent the mean of 3 independent experiments +/- SEM. * = p<0.05, ** = p <0.01 compared to vehicle-treated cells as determined by two-way ANOVA with Bonferroni post-hoc test. G) Growth in wild-type and acat-1-/- macrophages treated with or with T863 (10 µM). Error bars represent the mean of 3 independent experiments +/- SEM. ** = p <0.01, *** = p<0.001 as determined by two-way ANOVA with Bonferroni post-hoc test. H) Growth in vehicle and atglistatin-treated wild-type MH-S macrophages. Error bars represent the mean of 3 independent experiments +/- SEM., *** = p <0.001 as determined by two-tailed paired t-test. (TIFF) [file pone.0192215.s003.tiff]
