# Supplementary material for: Altering lipid droplet homeostasis affects Coxiella burnetii intracellular growth
Source: PLoS One. 2018 Feb 1;13(2):e0192215. doi: 10.1371/journal.pone.0192215 (PMC5794150; doi:10.1371/journal.pone.0192215)
Supplement: S1 File — This file describes the materials and methods for S1 Fig and S3 Fig. (DOCX) [file pone.0192215.s004.docx]

**Quantitative analysis of LD-associated proteins**

To determine PLIN2 and ACAT1 protein levels, 10^5^ cells/well were infected for 1 hour in a 24 well plate, at day 2 post-infection cells were lysed in 2% SDS (Sigma-Aldrich, St. Louis, MO, USA) for SDS‐PAGE and immunoblotting with 1:1000 rabbit anti-mouse ACAT1-specific antibody (Catalog # NBP189285, Novus Biologicals, Littleton, CO, USA), 1:1000 rabbit anti-mouse PLIN2 primary antibody (Catalog # PA1-16972, ThermoFisher Scientific) and 1:4000 GAPDH loading control monoclonal antibody (Catalog # MA5-15738, ThermoFisher Scientific, Waltham, MA, USA), and 1:10000 Donkey anti-mouse IRDye*680 (Catalog # P/N 925-68072, LI-COR biosciences, Lincoln, NE), 1:10000 Donkey anti-rabbit IRDye*800 (P/N 925-32213). Quantification was performed using Odyssey imaging systems (LI-COR biosystems, Lincoln, NE). Densitometry signal for each protein was normalized to respective GAPDH loading control and fold change over mock-infected cells was plotted.

***C. burnetii* growth by colony forming unit (CFU) assay**

To measure growth of *C. burnetii* in wild-type and *acat-1^-/-^* MH-S cells, 5x10^4^ cells/well were infected for 1 hour in a 48 well plate, washed with PBS, and then incubated with media containing respective vehicle and inhibitors. At the indicated time points, the media was removed and cells were incubated with sterile water for 5 min, pipetted up and down and the lysate diluted 1:5 in ACCM-2 + Tryptophan [1]. Serial dilutions were plated in triplicates on ACCM-2 + Tryptophan 0.25% w/vol agarose plates [1], incubated for 9-10 days, and colonies were counted. Each independent experiment was performed in duplicate.

**References**

1. Vallejo Esquerra E, Yang H, Sanchez SE, Omsland A. Physicochemical and Nutritional Requirements for Axenic Replication Suggest Physiological Basis for *Coxiella burnetii* Niche Restriction. Frontiers in cellular and infection microbiology. 2017;7:190. doi: 10.3389/fcimb.2017.00190. PubMed PMID: 28620582; PubMed Central PMCID: PMCPMC5449765.
